# Lumbosacral Radiculopathy as the Clinical Presentation of Neurocysticercosis

**DOI:** 10.4269/ajtmh.19-0757

**Published:** 2020-06

**Authors:** María Asunción Pérez-Jacoiste Asín, Patricia Calleja-Castaño, Amaya Hilario

**Affiliations:** 1 Unit of Infectious Diseases, Department of Internal Medicine, Hospital Universitario 12 de Octubre, Madrid, Spain;; 2 Department of Neurology, Hospital Universitario 12 de Octubre, Madrid, Spain;; 3 Department of Radiology, Hospital Universitario 12 de Octubre, Madrid, Spain

**Figure 1.**
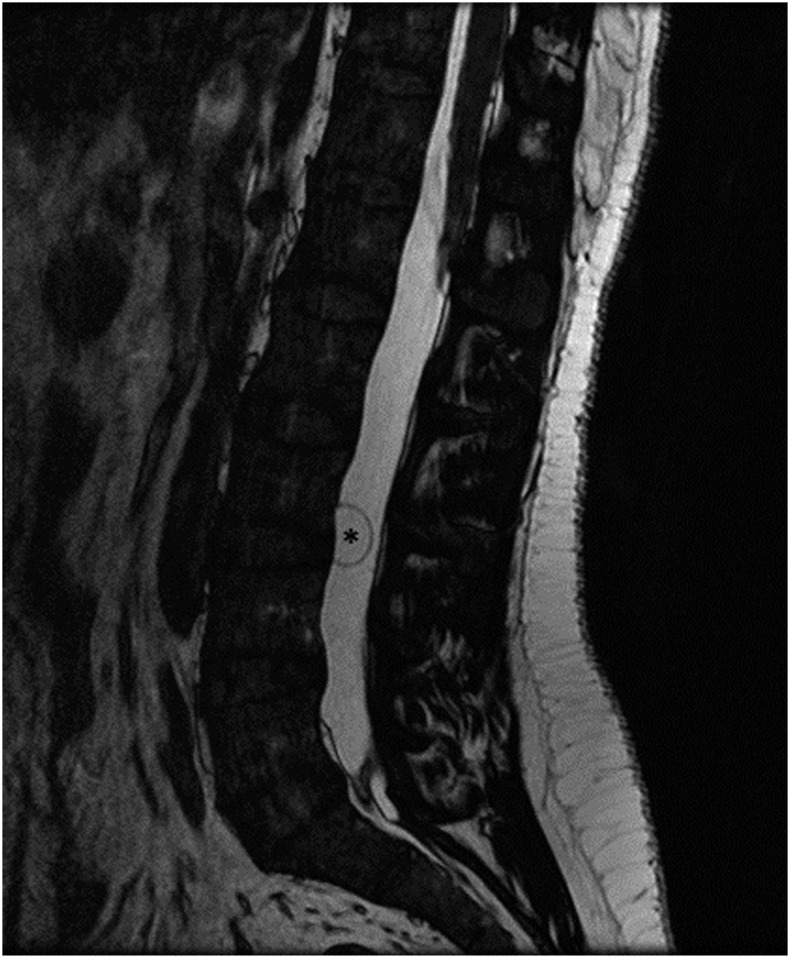
Spinal MRI: volumetric T2-weighted imaging in the sagittal plane shows a large intradural extramedullary cyst behind the L3 vertebral body (asterisk).

**Figure 2.**
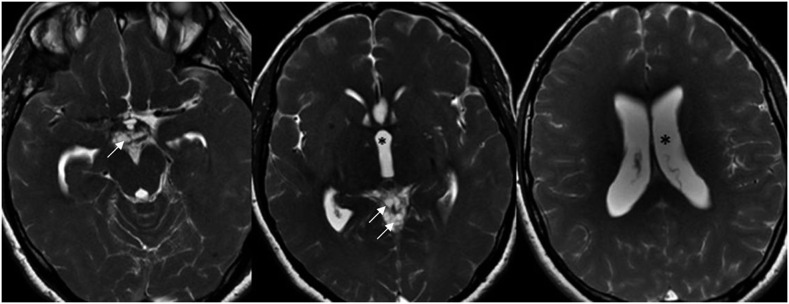
Brain MRI volumetric T2-weighted images. Hydrocephalus (asterisk) secondary to multiple cysts involving the suprasellar region and the supravermian cistern (arrows).

**Figure 3.**
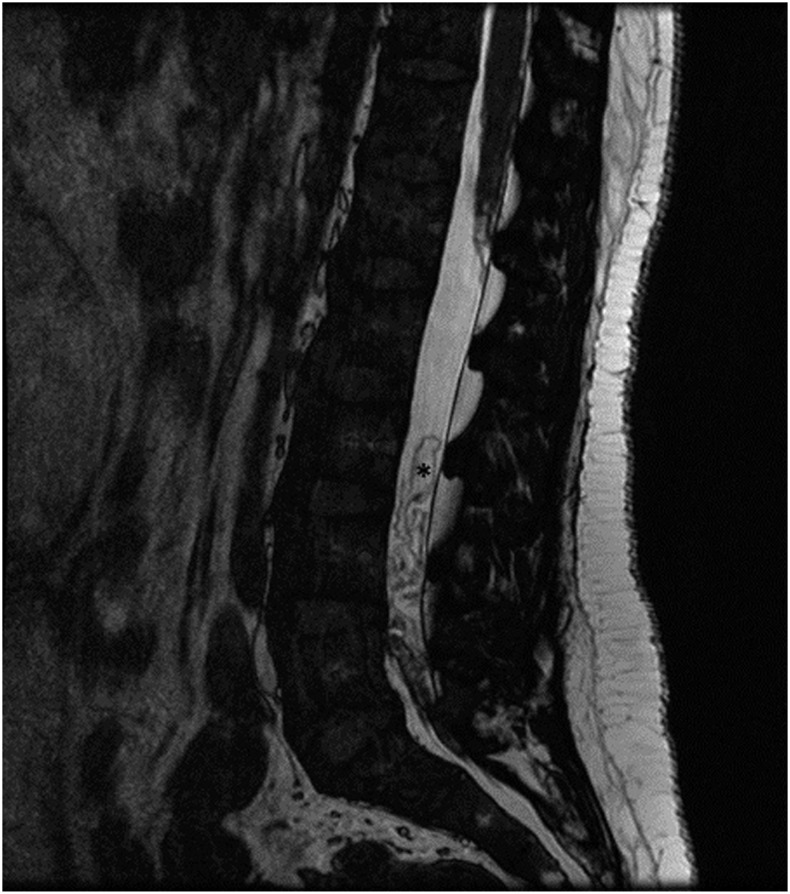
Spinal MRI after antiparasitic treatment; volumetric T2-weighted imaging in the sagittal plane shows clustered membranes resembling the folding of cysts (asterisk).

A 31-year-old woman from Ecuador, living in Spain since 2003, presented to our hospital in November 2017 complaining of disabling low back pain, which radiated into buttocks and lateral thighs, for 6 weeks. Spinal magnetic resonance image (MRI) demonstrated multiple intradural extramedullary cysts, the largest behind the L3 vertebral body, displacing cauda equina roots (Figure 1). With the suspicion of spinal neurocysticercosis, a diagnostic work-up was completed. Results were positive for cysticercosis serology, using a commercial ELISA to detect serum IgG antibodies to *Taenia solium* antigens, and also for the presence of *T. solium* HP10 antigen in serum, identified with a monoclonal antibody–based ELISA. Brain MRI confirmed numerous subarachnoid cysts involving basal cisterns and communicating hydrocephalus (Figure 2). When specifically asked, she noted mild headache for months. A ventriculoperitoneal shunt was placed. The analysis of the cerebrospinal fluid revealed an elevated protein concentration (1.21 g/dL) and positivity of *T. solium* HP10 antigen. After resolution of hydrocephalus, antiparasitic therapy was started, preceded by prednisone to control the inflammation. Because there were multiple subarachnoid cysts, she received a combination of albendazole and praziquantel for 4 weeks. Response was favourable, with persistence of mild lumbar pain, probably due to arachnoiditis, controlled with anti-inflammatory drugs. Serum *T. solium* antigen became negative and follow-up spinal MRI, performed 6 months later, showed clustered membranes resembling the folding of cysts after degeneration (Figure 3). After 2 years, she remained stable without symptoms or signs of recurrence.

Neurocysticercosis results from the ingestion of *T. solium* eggs shed in the stool of a human tapeworm carrier. After ingestion, oncospheres invade the intestine wall and spread hematogenously mainly to the central nervous system and muscles. Spinal neurocysticercosis occurs in 2% of all neurocysticercosis cases, and it is rare as an isolated condition.^1^ When spinal subarachnoid cysts are detected, brain MRI is mandatory because of the strong association with basilar subarachnoid involvement.^2^ Management of spinal neurocysticercosis should be individualized, based on symptoms, location of the cysts, degree of arachnoiditis, and surgical experience.^3^ The present report is an example that medical therapy with antiparasitic agents given concomitantly with corticosteroids may be successful for subarachnoid disease of the spine. Nevertheless, combination with surgical treatment could be necessary in some cases.
